# Highly Efficient Production of Soluble Proteins from Insoluble Inclusion Bodies by a Two-Step-Denaturing and Refolding Method

**DOI:** 10.1371/journal.pone.0022981

**Published:** 2011-07-29

**Authors:** Zhong Yang, Linlin Zhang, Yan Zhang, Ting Zhang, Yanye Feng, Xiuxiu Lu, Wenxian Lan, Jufang Wang, Houming Wu, Chunyang Cao, Xiaoning Wang

**Affiliations:** 1 State Key Laboratory of Genetic Engineering, Department of Microbiology, School of Life Sciences, Fudan University, Shanghai, China; 2 State Key Laboratory of Bioorganic and Natural Products Chemistry, Shanghai Institute of Organic Chemistry, CAS, Shanghai, China; 3 State Key Laboratory of Bioreactor Engineering, School of Biotechnology, East China University of Science and Technology, Shanghai, China; 4 School of Bioscience and Bioengineering, South China University of Science and Technology, Guangzhou, China; University of Oulu, Germany

## Abstract

The production of recombinant proteins in a large scale is important for protein functional and structural studies, particularly by using *Escherichia coli* over-expression systems; however, approximate 70% of recombinant proteins are over-expressed as insoluble inclusion bodies. Here we presented an efficient method for generating soluble proteins from inclusion bodies by using two steps of denaturation and one step of refolding. We first demonstrated the advantages of this method over a conventional procedure with one denaturation step and one refolding step using three proteins with different folding properties. The refolded proteins were found to be active using in vitro tests and a bioassay. We then tested the general applicability of this method by analyzing 88 proteins from human and other organisms, all of which were expressed as inclusion bodies. We found that about 76% of these proteins were refolded with an average of >75% yield of soluble proteins. This “two-step-denaturing and refolding” (2DR) method is simple, highly efficient and generally applicable; it can be utilized to obtain active recombinant proteins for both basic research and industrial purposes.

## Introduction

The over-expression of recombinant proteins in *Escherichia coli* (*E. coli*) is widely used to produce proteins in large amounts, due to noticeable advantages such as growth on inexpensive carbon sources, rapid biomass accumulation, amenability to high cell-density fermentation and relative ease for increasing production scale [1]–[4]. However, according to the statistics in the Center for Eukaryotic Structure Genomics (CESG) (http://targetdb.pdb.org/statistics/sites/CESG.html), among 8048 cloned targets in *E. coli*, only about 30% of them were expressed in soluble forms, whereas the others either were degraded or formed insoluble aggregates, known as inclusion bodies. Although inclusion bodies cannot be directly used for studies of protein activities, their insolubility provides an easy source of relatively pure protein, if only such proteins can be converted to their native and active conformation.

To obtain soluble active proteins from inclusion bodies, the insoluble inclusion bodies need to be first solubilized in denaturant, and then followed by a step of refolding process (for comparison, we refer to it here as “the one-step-denaturing and refolding” method) [5]. This procedure has been used over 20 years and works quite well for many inclusion body proteins, with approximately 40% being refolded to soluble and biologically active forms [5]–[7]. In this procedure, the inclusion bodies are denatured one time by using denaturing buffer containing either 6 M guanidine hydrochloride (GdnHCl), 8 M urea, or 0.3% sarkosyl (n-lauroyl sacosinate etc.). However, in most cases, there is a significant amount of precipitation when refolding the proteins, resulting in a great loss of overall yield of the target proteins.

We previously invented a new procedure called two-step-denaturing and refolding (2DR) method, which was originally used in 1998 to produce soluble rhG-CSF protein in a large scale efficiently with high quality for injection [8]. This was the first time that two denaturing steps (first GdnHCl and then urea) were used in the same procedure. In 2005, we modified the procedure for the hIL-2/GM-CSF inclusion body protein, with the following three steps: (1) Denaturation with 7 M GdnHCl, (2) Removal of GdnHCl by dialysis in 10 mM HCl buffer, (3) Addition of 8 M urea into the solution. In this modified 2DR procedure, 10 mM HCl was added to maintain hIL-2/GM-CSF in the soluble form during GdnHCl removal [9]. Recently, we developed a new 2DR procedure, which used an alkaline solution (pH>12) containing L-arginine as the first denaturant instead of GdnHCl, and used it to successfully refold four proteins to their soluble forms [10]. In 2009, we formulated a mathematical model of refolding protein to estimate the yield of soluble proteins from inclusion bodies by using 2DR method [11]. At the same time, we used the Harrison's two-parameter prediction model [12] to predict the solubility of 43 proteins refolded from inclusion bodies, and showed by 2DR experiments that this model could predict the refolding efficiency of inclusion bodies [13]. However, our previous studies did not compare the 2DR method with the conventional one-step-denaturing and refolding method regarding the quality and efficiency of protein refolding. Moreover, it was not clear whether the 2DR method is generally applicable.

Hence, in this study, we compared our 2DR procedure with the traditional one-step-denaturing and refolding method for protein refolding from inclusion bodies. We chose three representative proteins with different folding properties to compare the two methods, which are enhanced green fluorescent protein (EGFP), the catalytic domain of human macrophage metalloelastase (MMP-12), and the DNA binding domain of neuronal restricted silencing factor/RE-1 silencing transcription factor (NRSF/REST DBD). Furthermore, to investigate whether the 2DR technique can be utilized broadly on other inclusion body proteins, we analyzed 88 insoluble inclusion body proteins for refolding to their soluble forms by the 2DR technique.

## Results

### A brief description of the two-step-denaturing and refolding (2DR) method

The first denaturing step was to thoroughly dissolve inclusion bodies, using a denaturing buffer with 7 M GdnHCl (*i.e.* extraction buffer I). Subsequently, to precipitate the GdnHCl-denatured protein, the protein solution was diluted by the dilution buffer. The second denaturing step was to dissolve the protein precipitate by a denaturing buffer with 8 M urea (*i.e.* extraction buffer II). Then the refolding of the target protein was conducted either on a column, or by drop-wise dilution, or by stepwise dialysis, as described before [7]. Purification and characterization of target proteins were then carried out by using high-resolution ion-exchange chromatography and SDS-PAGE gel, respectively. The conformation of the refolded proteins were monitored by acquiring circular dichroism (CD) or NMR two-dimensional ^1^H-^15^N HSQC spectrum; the aggregation-states of the refolded target proteins were analyzed by either running dynamic light scattering (DLS) experiment, or running non-denaturing PAGE gel, or using size exclusion chromatography.

### Rationale for choice of three representative proteins

To test quality and efficiency of refolding by the 2DR method and to compare with the one-step denaturing and refolding method, we analyzed three proteins with different folding properties: EGFP, MMP-12, and NRSF/REST DBD (Figure S1). EGFP was selected as a reference protein, because it could be produced in both soluble form and insoluble inclusion bodies from *E. coli* over-expression system, depending on culturing conditions [14]–[15]. In addition, the green fluorescence can be used as a sensitive assay to monitor protein folding [15]. EGFP samples from soluble expression or refolding of inclusion bodies using either one-step or two-step denaturing and refolding techniques could be examined for possible difference in Circular Dichroism (CD) and fluorescence spectrometry. MMP-12 was reported to be over-expressed in *E. coli* only as inclusion bodies, and its active form could be obtained by using the one-step denaturing and refolding method [16]–[18]. So, we can test whether soluble and active MMP-12 could be obtained by using the 2DR method and compare the differences in the overall yield and biological activity of soluble MMP-12 between one-step and two-step denaturing techniques. NRSF/REST plays a critical role in neuronal gene expression of the central nervous system (CNS) by specifically binding to neuron restrictive regulatory elements [19]–[21]. It is important to obtain soluble form of the DNA-binding domain (DBD), which contains eight tandem zinc fingers and interacts with nucleic acids [21], for detailed functional and structural studies. However, there is no reported method to obtain soluble DBD from *E. coli* over-expression systems. Our preliminary analysis indicated that DBD expressed in *E. coli* formed inclusion bodies and could not be refolded to soluble and biologically active form by the one-step denaturing and refolding method. Therefore, DBD was selected as a representative example to demonstrate the advantages of 2DR method over one-step denaturing process.

### Highly efficient production of 3 soluble proteins by 2DR method

Both soluble form and inclusion bodies of EGFP were expressed in *E. coli*. Using the 2DR method, 28 mg of pure and soluble EGFP was successfully refolded from its inclusion bodies; this yield was three times as much as the 9 mg obtained through the one-step denaturing and refolding procedure, and comparable to 33 mg of EGFP produced by soluble expression in *E. coli*. The refolding yield was defined as the percentage of soluble proteins in the refolding buffer over the amount of denatured proteins in extraction buffer II. Because no precipitation was detected in the refolding buffer, the overall yield of soluble EGFP was nearly 100%.

**Figure 1:**
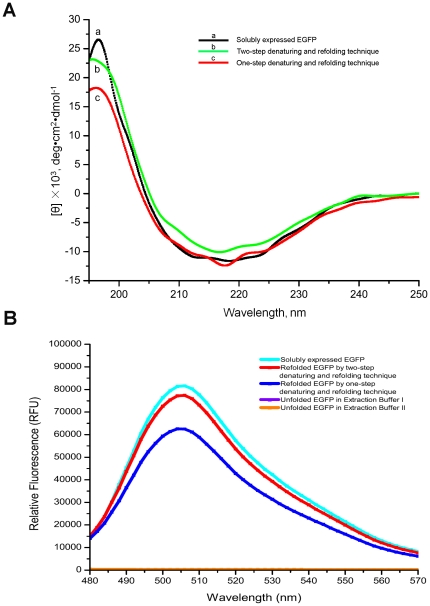
The different behaviors of EGFP were displayed on (A) CD spectra and (B) fluorescence spectra.

**Figure 2:**
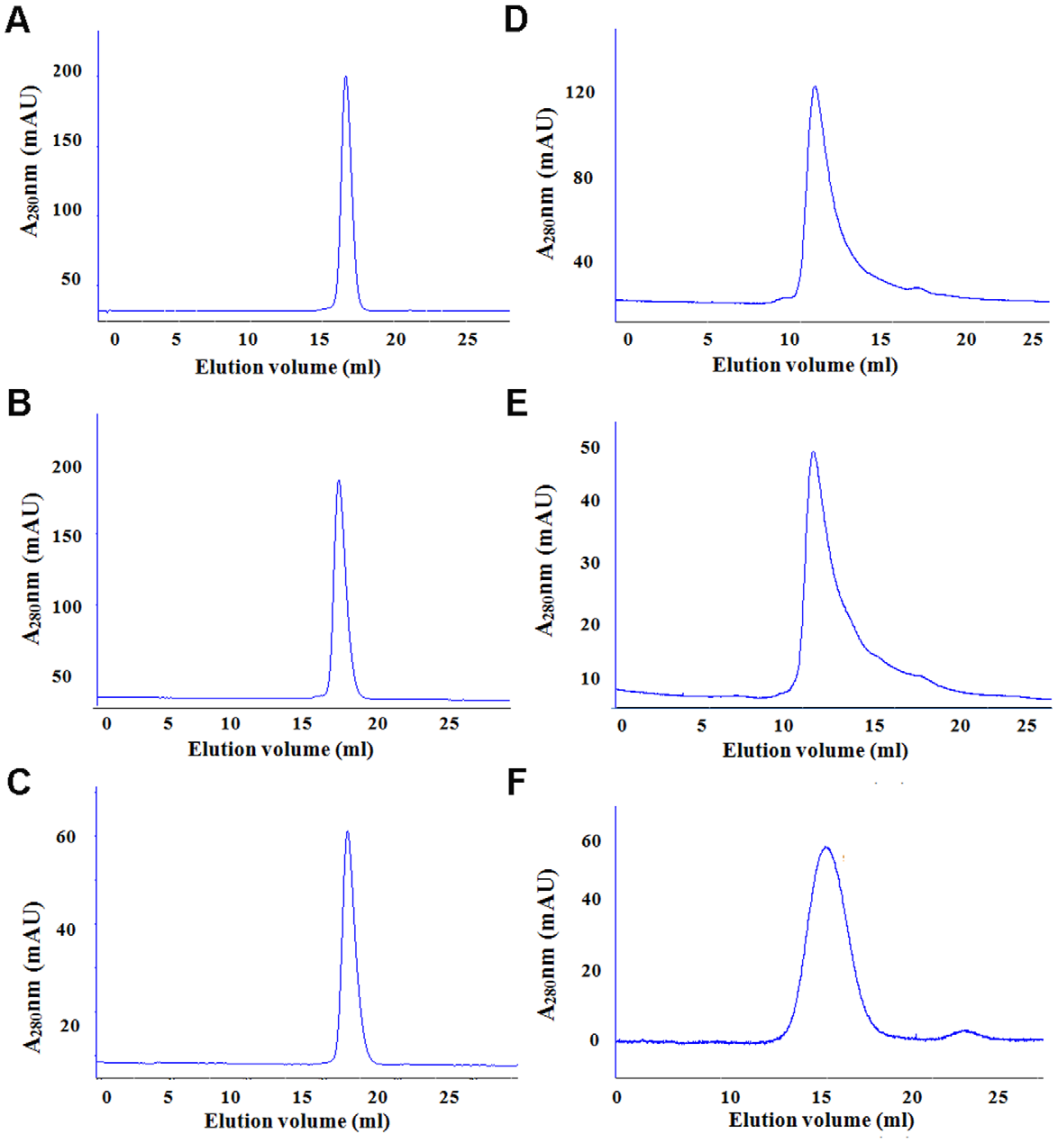
Size exclusion chromatography running at a Superdex-200 column for EGFP, refolded MMP-12 and refolded DBD.

As shown in Figure 1, the maximal fluorescent intensities at 506 nm were 8162 RFU for the solubly expressed EGFP, 7738 RFU for EGFP from 2DR, 6246 RFU for EGFP from one-step denaturing and refolding, and 269 RFU for unfolded EGFP in extracting buffer II, all at the concentration of 1 mg/ml. Therefore, the fluorescence absorption at 506 nm of refolded EGFP through 2DR technique is close to that of EGFP from soluble expression (94.8%), higher than that of 76.5% of refolded EGFP through the one-step-denaturing and refolding procedure. These fluorescence behaviors were consistent with the results from CD spectra, where refolded EGFP generated using 2DR procedure was predicted to have 22.7% (an average of three experiments, with a range of 21–25.7) of helical structures, close to that (32.7%) of solubly expressed EGFP, but much higher than that (2.8%) of refolded EGFP through one-step denaturing and refolding technique. The latter form of EGFP even had 30% of random structure. In addition, size exclusion chromatography demonstrated that all EGFP samples had almost the same elution volume (about 16 ml) (Figure 2), indicating that three EGFP samples were all monomers instead of aggregates.

MMP-12 was expressed in *E. coli* as inclusion bodies, and 45 mg of soluble form was produced through the 2DR method, almost double that the amount (23 mg) obtained from one-step denaturing and refolding method [18]. During the refolding step, there was no precipitation observed, so the refolding yield of MMP-12 was also close to 100%. The biochemical activity of the refolded MMP-12 was measured as *k*_cat_/*K*_m_ at 25°C is 2.5×10^5^ M^−1^S^−1^, comparable with the literature value 1.5×10^5^ M^−1^S^−1^ of the refolded MMP-12 obtained using the method of one-step denaturing and refolding [17]–[18]. Size exclusion chromatography indicated that the two refolded soluble MMP-12 proteins obtained from one-step and two-step denaturing and refolding techniques, respectively, had same elution volume of about 11 ml, suggesting that both were monomers (Figure 2).

**Figure 3:**
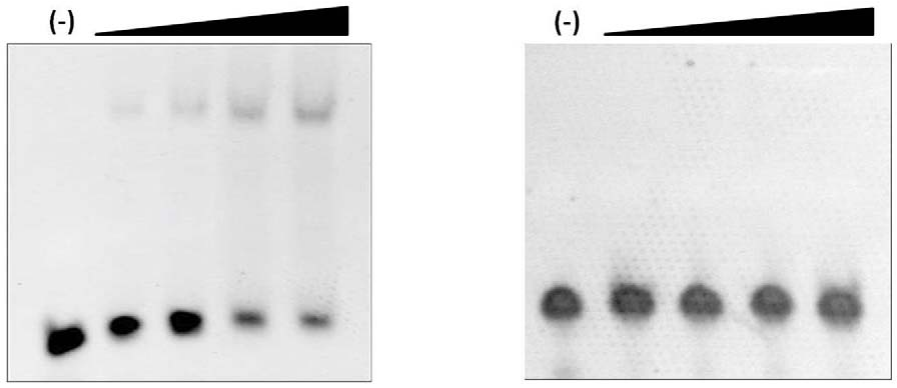
Gel shift binding assay of the refolded (left pane) and unfolded (right pane) NRSF/REST DBD.

The DBD fragment of NRSF/REST was over-expressed in *E. coli* as inclusion bodies. To obtain soluble NRSF/REST DBD from the inclusion bodies, we first use the one-step denaturing and refolding technique. After several attempts, we could not obtain any soluble DBD protein. In contrast, we successfully refolded DBD to its soluble and biologically active form using the 2DR technique and obtained 7 mg of pure and soluble protein. The result from size exclusion column demonstrated that DBD was monomer, with about 15 ml of elution volume (Figure 2). To probe whether the refolded DBD has activity, we detected its binding affinity to NRSE/RE1 dsDNA by using native DND PAGE gel shift binding assay [22], using unfolded DBD as negative control (Figure 3). The native gel binding assay indicated that the refolded DBD had binding affinity to NRSE/RE1 dsDNA, while unfolded DBD did not.

In summary, the comparison studies of EGFP, MMP-12 and DBD demonstrated that the refolding yield with 2DR technique is significantly better than the one-step denaturing and refolding method.

### General applicability of the 2DR method

To further investigate the versatility of the 2DR technique, we refolded 88 inclusion body proteins collected from different laboratories in China. Among 88 inclusion body proteins (Table S2), 27% of them were refolded to their soluble forms without any precipitation (after centrifuging at 30,000 g) observed during refolding, so the soluble refolding yield of these proteins was approximately 100%; 40% of the proteins were refolded with yields between 90% and 100%, and 9% of them with yields of 75–90%. Only 13.6% of them were refolded to soluble form with yields less than 50%. Accordingly, the yield of soluble proteins refolded from inclusion bodies with more than 75% yield was approximately 76%. These data suggested that the 2DR technique can be widely applied to refold insoluble inclusion body proteins with high efficiency.

## Discussion

In the one-step-denaturing and refolding process, either 6 M GdnHCl or 8 M Urea denaturant is most commonly used to solubilize the inclusion bodies in denature form [5]. Then the denaturant should be reduced and removed from the solubilized protein solution by dilution or dialysis in a huge volume of refolding buffer to recover soluble and active proteins. In this step, protein folding/unfolding intermediates are usually formed; these intermediates often aggregate due to contiguous hydrophobic groups [23]–[24]. This process of protein aggregation often leads to a significant amount of protein precipitation, a great reduction of overall yield of the target proteins.

In contrast, the 2DR technique emphasizes two steps of denaturing; the first GdnHCl denaturing is to completely unfold the misfolded protein in the inclusion bodies. However, while GdnHCl is being removed, protein precipitation often occurs; the second urea denaturing is designed to dissolve these protein precipitates. From our analyses of 88 inclusion body proteins, a key aspect of the 2DR method is to precipitate the GdnHCl-denatured protein through dilution (especially rapid dilution), such that as much precipitation as possible is achieved to reducing the loss of protein in the supernatant. We also found that, if the GdnHCl-denatured protein precipitation can be dissolved by the second urea denaturing buffer, most likely high yielding soluble proteins can be obtained.

According to the previous report [25]–[26], the inclusion bodies formed in *E. coli* contain misfolded secondary protein structures, which then lead to protein aggregation during the refolding process of solubilizing inclusion bodies. We speculate that the first step of strong denaturant GdnHCl of the 2DR method can unfold these misfolded secondary structures completely into random coiled structures. After rapid dilution of the solution containing solubilized inclusion body protein, nearly homogeneous partially folded intermediates might be formed as the precipitates. When the precipitates are resolubilized by relatively mild urea denaturant, the homogeneous partially folded intermediates would allow formation of correct structures in the subsequent protein folding process. Thus, further efforts in the feature will focus on monitoring the conformation dynamic of protein folding pathway in the 2DR method to determine the possible mechanism of our technique.

In conclusion, this study has shown that the 2DR method is more efficient than the traditional one-step-denaturing and refolding method, using the representatives EGFP, MMP-12 and DBD proteins with different folding properties. Furthermore, the 2DR method is simple and generally applicable for diverse insoluble proteins, it might be utilized to obtain active recombinant proteins for both basic research and industrial purposes.

## Materials and Methods

### EGFP soluble expression and purification

The gene encoding EGFP was cloned into pTO-T7 expression vector [27], then the plasmid was transferred into BL21 (DE3) CodonPlus strain (Stratagene). The cells were grown in 1 liter of LB medium at 37°C, and when the absorbance at 600 nm (OD_600_) reached 0.7–0.8, a final concentration 1 mM of isopropyl-β-D-thiogalactopyranoside (IPTG) was added to induce the expression at 16 °C for another 18 hours, then the cells were harvested at 6,000 g at 4°C for 15 minutes and resuspended in 40 ml of buffer A (50 mM Tris-HCl, 50 mM NaCl, 5 mM β-mercaptoethanol, pH 8.0). The mixture was stirred continuously with magnetic stir bar for 1 hour at 4°C, and then lysed by sonication on ice. The lysate was centrifuged at 30,000 g at 4°C for 45 minutes and the resulting clarified supernatant was collected and loaded onto a Macro-prep High Q ion exchange column (10 ml in size, BioRad) previously equilibrated with 50 ml of buffer A. Subsequently, the protein was eluted out by using a linear salt gradient from 150 mM–1 M NaCl by using buffer B (50 mM Tris-HCl, 1 M NaCl, 5 mM β-mercaptoethanol, pH 8.0). The EGFP contained fractions were pooled together and then purified on a DEAE column (10 ml in size, Bio-Rad) with the two buffers same to those used on High Q column above. The fractions including EGFP were concentrated again by using a YM-10 membrane (Amicon), and then loaded onto a gel filtration Superdex-200 column (HiLoad™ 16/60, GE Healthcare Inc.) equilibrated with five columns of buffer A. Purified EGFP was eluted out with buffer A at a flow rate of 2 ml/min, and confirmed by SDS-PAGE gel analysis (Figure S2).

### EGFP over-expression in *E. coli* as inclusion bodies

Using the same EGFP pTO-T7 plasmid and BL21(DE3) CodonPlus strain above, cells were grown to OD_600_ of 0.7–0.8; then 1 mM IPTG was used to induce EGFP expression at 37°C for 4 hours. The cells were harvested at 6,000 g at 4°C for 15 minutes, washed twice with 150 ml of washing buffer I (20 mM Tris, pH 8.0), centrifuged (30,000 g, 4°C, 30 minutes), lysed with 80 ml of buffer C (50 mM Tris, pH 8.0) by sonication on ice. The lysate was centrifuged (30,000 g, 4°C, 45 minutes). The pellets were cleaned twice with 150 ml of washing buffer II (50 mM Tris, 50 mM NaCl, 2% Triton X-100, 1.5 mM β-mercaptoethanol, 1.6 M urea, pH 8.0), the mixture solution was stirred for 20 minutes, centrifuged (30,000 g, 4°C, 45 minutes). To remove Triton X-100, the inclusion bodies were further cleaned two more times with 150 ml of washing buffer I (20 mM Tris, pH 8.0). Finally, the pellets were kept at −20°C.

### One-step denaturing and refolding of EGFP inclusion bodies

The isolated inclusion bodies were dissolved in 5 ml of extraction buffer II (50 mM Tris, 50 mM NaCl, 10 mM β-mercaptoethanol, 8 M urea, pH 8.0), the solution was stirred for about 20 minutes and centrifuged (30,000 g, 4°C, 45 minutes), the supernatant was collected and pellets were discarded. The total protein concentration in this stage was measured by using the Bradford method [28] and adjusted to 8–10 mg/ml. Then the solution was added drop-wise into 400 ml of refolding buffer (20 mM Tris, 1 mM EDTA, 1 mM GSH, 0.1 mM GSSG, pH 8.0) and stirred slowly with magnetic stir bar at 4°C for two days, then loaded at a rate of 0.5 ml/min onto a Macro-prep High Q column (Bio-rad) balanced with 50 ml of refolding buffer. The protein was eluted by using a linear salt gradient from 150 mM–1 M NaCl in buffer D (20 mM Tris, 1 mM EDTA, 1 M NaCl, pH 8.0), the fractions containing EGFP were confirmed by running SDS-PAGE gel (Figure S2), concentrated with a YM-10 membrane (Amicon), and the amount of EGFP was determined by measuring A^280^, and by using extinction coefficient constant of 20,400 M^−1^ cm^−1^ at 280 nm (Thermo, He<$>\vskip -1.1\scale55%\raster="rg1"<$>ios γ). Finally, to analyze the oligomeric state of EGFP, concentrated EGFP fractions were loaded onto a Superdex-200 column for size exclusion chromatography (Superdex™ 10/300GL, GE Healthcare Inc.) poised with buffer A (50 mM Tris-HCl, 50 mM NaCl, 5 mM β-mercaptoethanol, pH 8.0) at a flow rate of 0.5 ml/min (Figure 2).

### Two-step denaturing and refolding of EGFP inclusion bodies

The cleaned inclusion bodies were dissolved in 5 ml of extraction buffer I (50 mM Tris, 50 mM NaCl, 10 mM β-mercaptoethanol, 7 M GdnHCl, pH 8.0), then the solution was centrifuged (30,000 g, 4°C, 30 minutes). The supernatant was diluted into 200 ml of dilution buffer (50 mM Tris, 1 mM EDTA, 50 mM NaCl, 10 mM β-mercaptoethanol, pH 8.0) to rapidly precipitate denatured EGFP, followed by centrifugation (30,000 g, 4°C, 45 minutes). The pellets were collected, and dissolved in 5 ml of extraction buffer II (50 mM Tris-HCl, 50 mM NaCl, 10 mM β-mercaptoethanol, 8 M urea, pH 8.0). The details for further refolding, purification and oligomeric state determination (Figure 2) of refolded EGFP were conducted in the same way to those done on refolded EGFP by using one-step denaturing and refolding technique, as described above.

### Fluorescent spectra on different EGFP samples

As shown in Figure 1, to investigate the effects on the EGFP refolding of different denaturing techniques, the fluorescence spectra of four EGFP samples, including unfolded EGFP (negative control), solubly expressed EGFP, two refolded EGFP from inclusion bodies by using one-step or two-step denaturing and refolding techniques, were adjusted to concentration of 1.0 mg/ml. Excitation was set at 450 nm and emission spectra at 480–570 nm were recorded using a fluorescent spectrometer SpectraMax M5 (Molecular Devices Inc.). Slit width was set at 2.5/1 nm. In Figure 1B, two unfolded EGFP samples dissolved in extracting buffer I and II were also used as negative control, which were overlapped into a horizontal line.

### Circular dichroism spectra of EGFP samples

To investigate the secondary structure contents of EGFP samples obtained from one-step or two step denaturing techniques, CD spectra were acquired on these samples with J-715 spectropolarimeter (Jasco), using solubly expressed EGFP as a control. The photomultiplier voltage read never exceeded 600 V in the spectral regions. Each spectrum was averaged from five measurements and smoothed with spectropolarimeter system software Jascow32. All measurements were performed at room temperature under a nitrogen flow. The protein concentration was 50 µM. CD spectra were recorded in a 1 mm pathlength cell from 190 to 250 nm with a step size of 0.1 nm, a bandwidth of 1 nm. CD spectrum of the appropriate buffer (1 mM Tris, pH 8.0) was recorded and subtracted from the protein spectra. To obtain the α-helix content of EGFP, all CD data was analyzed by using the method of Least Squares Minimization [29]–[30].

### MMP-12 inclusion bodies generation

The gene coding for human MMP-12 was cloned into *BamH* I and *Nde* I sites of a T7-based expression plasmid pET11c vector (Novagen), then the expression plasmid was transferred into the strain BL21 (DE3). The cells were grown in 1 liter of LB medium at 37°C. When OD_600_ reached 0.7–0.8, protein over-expression was induced by IPTG with a final concentration of 1 mM at 37°C for another 4 hours, then the cells were harvested at 6,000 g at 4°C for 15 minutes, washed with 150 ml of washing buffer I, centrifuged (30,000 g, 4°C, 30 minutes), then lysed with 80 ml of buffer C by sonication on ice, centrifuged (30,000 g, 4°C, 45 minutes). The supernatant was discarded, and the pellets were stored at −20°C.

### Two-step denaturing and refolding of MMP-12 inclusion bodies

The inclusion bodies were first cleaned with 150 ml of washing buffer II, the resuspension solution was then centrifuged (30,000 g, 4°C, 30 minutes). Triton X-100 was removed by washing precipitated denatured MMP-12 two times with 150 ml of washing buffer I, as did for EGFP inclusion bodies. Then the inclusion bodies were dissolved in 5 ml of extraction buffer I, centrifuged (30,000 g, 4°C, 30 minutes). The supernatant was diluted into 200 ml of dilution buffer. At this point, precipitation was observed, and was collected by centrifuging (30,000 g, 4°C, 10 minutes). The precipitation was further dissolved in 5 ml of extraction buffer II, then loaded onto a Sepharose Q column (5 ml in size, G.E. Healthcare Inc.) that was previously equilibrated with 25 ml of buffer E (50 mM Tris, 5 mM β- mercaptoethanol,6 M Urea,pH 8.0). A gradient was run from buffer E to buffer F (50 mM Tris, 5 mM β- mercaptoethanol,6 M Urea,1 M NaCl, pH 8.0). The MMP-12 fractions were confirmed by running SDS-PAGE gel, collected. To avoid precipitation shown up, the concentration of MMP-12 was adjusted to 0.1 mg/ml, and then placed into a MWCO 3 K dialysis membrane, and then refolded in 5 liters of refolding buffer (20 mM Tris, 100 mM NaCl, 10 mM CaCl_2_, 0.2 mM ZnCl_2_, pH 7.5) through equal-size stepwise dialysis (Figure S3). In this step, the refolding buffer and denaturing buffer G (50 mM Tris, 6 M Urea, 100 mM NaCl, 10 mM CaCl_2_, 0.2 mM ZnCl_2_, pH 7.5) were pooled in the bottles A and B, respectively. The dialysis membrane containing MMP-12 fractions was placed in bottle B. The flow rates of refolding buffer from bottle A into bottle B, and of denaturing buffer G from bottle B into bottle C were equally set as 2.2 ml/min by using pump. After dialysis was done, the refolded MMP-12 solution was concentrated with a YM-10 membrane (Amicon). MMP-12 purity was confirmed with SDS-PAGE gel (Figure S4). The amount of MMP-12 was calculated by using A280 and extinction coefficient of 26930 M-1 cm-1 at 280 nm (Thermo, He
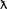
ios γ). Similarly, to investigate the oligomeric state of MMP-12, size exclusion chromatography was run at a Superdex-200 column (Superdex™ 10/300GL, GE Healthcare Inc.) at a flow rate of 0.5 ml/min as for EGFP (Figure 2).

### The biological activity of MMP-12 catalytic domain

The activity of MMP-12 refolded with two-step denaturing technique was measured by using the fluorescence assay, as done in reported literature [18]. It was performed in black 96-well plates (Corning) in a final volume of 100 µl at pH 7.4 containing: 50 mM Tris-HCl, 10 mM CaCl_2_, 0.05% Brij-35 (v/v), 10 µM substrate (Mca-Pro-Leu-Gly-Leu-Dpa-Ala-Arg-NH_2_ from CALBIOCHEM) and 1 nM MMP-12 at 25°C. The release of the fluorescent product McaPL was monitored continuously using a Genios Pro fluorescent reader, using 340 nm excitation/400 nm emission, and calibrated to a standard curve of this compound. Initial rates were calculated over a time period, typically 20 minutes, where the cleavage of substrate was linear with respect to time and did not exceed 10% conversion.

### DBD inclusion bodies generation

The DNA region coding for the mouse NRSF/REST DBD was obtained through PCR amplification, and cloned into the *BamH* I and *Nco* I sites of a T7-based pET15b expression plasmid (Novagen), which was further transformed into expression strain Rosetta 2(DE3) (Merck). The cells were grown in 1 liter of LB medium at 37°C, and the expression was also induced for 4 hours with 1 mM IPTG. The subsequent operations such as cells harvesting, washing, resuspending, and lysing, and Triton X-100′s removing were performed as similarly as those processed on MMP-12.

### Two-step denaturing and refolding of NRSF/REST DBD inclusion bodies

The operation details about how to denature NRSF/REST DBD inclusion bodies firstly with 7 M GdnHCl included extracting buffer I and secondly by 8 M urea contained extracting buffer II were identical with those for EGFP mentioned above. Then NRSF/REST DBD was refolded through stepwise dialysis. The dialysis buffer conditions were listed in Table S1. The refolded DBD solution was loaded onto a HP Heparin column (5 ml in size, G.E. Healthcare Inc) which was equilibrated with 25 ml of buffer H (20 mM Tris, 150 mM NaCl, 1 mM ZnCl_2_, 10 mM β-mercaptoethanol, pH 7.3), and the protein was eluted out through a linear salt gradient from 150 mM - 1 M NaCl by using buffer K (20 mM Tris, 1 M NaCl, 1 mM ZnCl_2_, 10 mM β-mercaptoethanol, pH 7.3). The identity of the protein was confirmed by using MALDI-TOF Mass Spectroscopy and by running SDS-PAGE gel (Figure S5). Similarly, to examine the aggregation state of NRSF/REST DBD, size exclusion chromatography was run on a Superdex-200 column (Superdex™ 10/300GL, GE Healthcare Inc.) at a flow rate of 0.5 ml/min as done for EGFP (Figure 2).

### Native DNA gel shift binding assay of DBD to the NRSE/RE1 dsDNA

Each strand of NRSE/RE1 dsDNA was commercially synthesized at HPLC grade (Shanghai Sangon Biological Engineering Technology and Service Co. Ltd, China) containing the following base sequences: 5′-TTC AGC ACC ACG GAC AGC GCC-3′ and 5′-GGC GCT GTC CGT GGT GCT GAA-3′. The DNA binding assay was performed using a fixed concentration of 75 pmol of annealed NRSE/RE1 dsDNA. The refolded DBD was added at the molar ratios of DBD to NRSE/RE1 dsDNA (0:1, 0.5:1, 1:1, 2:1, 3:1, respectively, as indicated from left to right in each pane of Figure 3). The mixed solution was incubated for 30 minutes at room temperature in the final volume of 20 ul in buffer condition: 10 mM Tris, pH 7.0, 0.1 mM ZnCl_2_. Then two native DNA PAGE gels (5% polyacrylamide contained) were run, which were further stained with EtBr to indicate the interaction results. The unfolded DBD was used as a negative control.

### Two-step denaturing and refolding of 88 inclusion bodies proteins

The plasmids containing the genes of 88 proteins were transferred into the BL21(DE3) strain. The cells were grown in 1 liter of LB medium at 37°C. All over-expressions were induced by 1 mM IPTG at 37°C for 4 hours. The cells were harvested at 6,000 g at 4°C for 15 minutes, washed twice with 150 ml of washing buffer I, centrifuged (30,000 g, 4°C, 30 minutes), lysed with 80 ml of buffer C by sonication on ice, and centrifuged (30,000 g, 4°C, 40 minutes). The pellets were cleaned twice with 150 ml of washing buffer II, centrifuged (30,000 g, 4°C, 30 minutes). Before being denatured, the pellets were cleaned two times with 150 ml of washing buffer I to remove Triton X-100. The detailed experimental operations of two-step denaturing and refolding of these inclusion bodies were identical to those conducted on EGFP. The purification steps of each protein were done by using different high resolution ion-exchange chromatography dependent on the chemical properties (such as pKa values) of the target proteins, the identities of these proteins were characterized by running SDS_PAGE gels and MALDI-TOF Mass spectroscopy. Size exclusion chromatography was used to evaluate their oligomeric states. The final results were summarized in the Table S2.

## Supporting Information

Figure S1 **Schematic diagrams of NRSF/REST and its fragment DBD construct mentioned in the paper,** ***boxes*** **indicating repression domains (RD1 and RD2 in both N and C termini) or Pro-rich domain,** ***standing ellipses*** **indicating zinc fingers,** ***lying ellipse*** **indicating Lys-rich region.**
(DOC)

Figure S2 **SDS-PAGE gel indicated EGFP production and purification during soluble expression (A), one-step denaturing and refolding (B) and two-step denaturing and refolding (C) of incluison bodies.** (A), line 1, Marker; lane 2, cells before IPTG induction; lane 3, cells after IPTG induction; lane 4, supernatant; lane 5, purified EGFP; (B) lane 1, cells before IPTG induction; lane 2, cells after IPTG induction; lane 3, inclusion bodies, lane 4, refolded EGFP, lane 5, marker; (C) lane 1, cells before IPTG induction; lane 2, cells after IPTG induction; lane 3, inclusion bodies, lane 4, refolded EGFP, lane 5, marker.
(DOC)

Figure S3 **The equipment of stepwise dialysis for MMP-12 refolding.**
(DOC)

Figure S4 **SDS-PAGE gel indicated that the catalytic domain of MMP-12 was recovered from its inclusion bodies by using double denaturing and refolding method.** From right to left, lane 1: the cells before IPTG introduction; lane 2, the cells after IPTG introduction; lane 3, MMP-12 inclusion bodies; lane 4,MMP-12 in denatured buffer II ; lane 5,refolded MMP-12; lane 6, protein marker. The molecular weight of the catalytic domain of MMP-12 is 18.5KDa.
(DOC)

Figure S5 **SDS-PAGE gel indicated DBD generation through two-step denaturing and refolding.** Lane 1, protein marker; lane 2, cells before IPTG induction; lane 3, cells after IPTG induction; lane 4, DBD inclusion bodies dissolved in the extracting buffer 2; lane 5, refolded DBD.
(DOC)

Table S1 **The gradient dialysis buffer for refolding DBD from denatured buffer II.**
(DOC)

Table S2 **Application of two-step denaturing and refolding technique on 88 different inclusion bodies.**
(DOC)
