# Supplementary material for: Highly Efficient Production of Soluble Proteins from Insoluble Inclusion Bodies by a Two-Step-Denaturing and Refolding Method
Source: PLoS One. 2011 Jul 29;6(7):e22981. doi: 10.1371/journal.pone.0022981 (PMC3146519; doi:10.1371/journal.pone.0022981)

**Figure S1**: Schematic diagrams of NRSF/REST and its fragment DBD construct mentioned in the paper, *boxes* indicating repression domains (RD1 and RD2 in both N and C termini) or Pro-rich domain, *standing ellipses* indicating zinc fingers, *lying ellipse* indicating Lys-rich region.


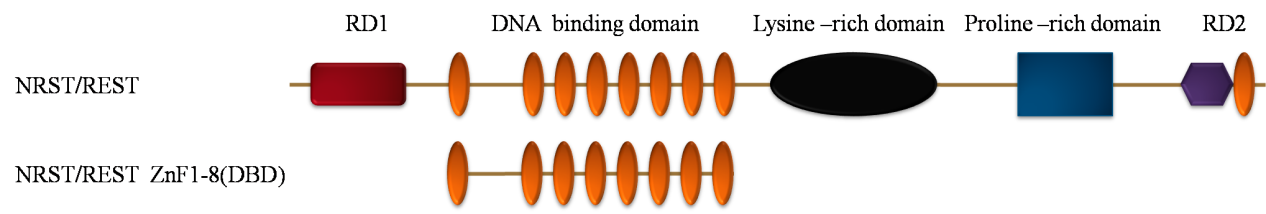

Supplement: Figure S1 — Schematic diagrams of NRSF/REST and its fragment DBD construct mentioned in the paper, boxes indicating repression domains (RD1 and RD2 in both N and C termini) or Pro-rich domain, standing ellipses indicating zinc fingers, lying ellipse indicating Lys-rich region. (DOC) [file pone.0022981.s001.doc]
