# Supplementary material for: Highly Efficient Production of Soluble Proteins from Insoluble Inclusion Bodies by a Two-Step-Denaturing and Refolding Method
Source: PLoS One. 2011 Jul 29;6(7):e22981. doi: 10.1371/journal.pone.0022981 (PMC3146519; doi:10.1371/journal.pone.0022981)
Supplement: Figure S2 — SDS-PAGE gel indicated EGFP production and purification during soluble expression (A), one-step denaturing and refolding (B) and two-step denaturing and refolding (C) of incluison bodies. (A), line 1, Marker; lane 2, cells before IPTG induction; lane 3, cells after IPTG induction; lane 4, supernatant; lane 5, purified EGFP; (B) lane 1, cells before IPTG induction; lane 2, cells after IPTG induction; lane 3, inclusion bodies, lane 4, refolded EGFP, lane 5, marker; (C) lane 1, cells before IPTG induction; lane 2, cells after IPTG induction; lane 3, inclusion bodies, lane 4, refolded EGFP, lane 5, marker. (DOC) [file pone.0022981.s002.doc]

**Figure S2:** SDS-PAGE gel indicated EGFP production and purification during soluble expression (A), one-step denaturing and refolding (B) and two-step denaturing and refolding (C) of incluison bodies. (A) , line 1, Marker; lane 2, cells before IPTG induction; lane 3, cells after IPTG induction; lane 4, supernatant; lane 5, purified EGFP; (B) lane 1, cells before IPTG induction; lane 2, cells after IPTG induction; lane 3, inclusion bodies, lane 4, refolded EGFP, lane 5, marker; (C) lane 1, cells before IPTG induction; lane 2, cells after IPTG induction; lane 3, inclusion bodies, lane 4, refolded EGFP, lane 5, marker.

**
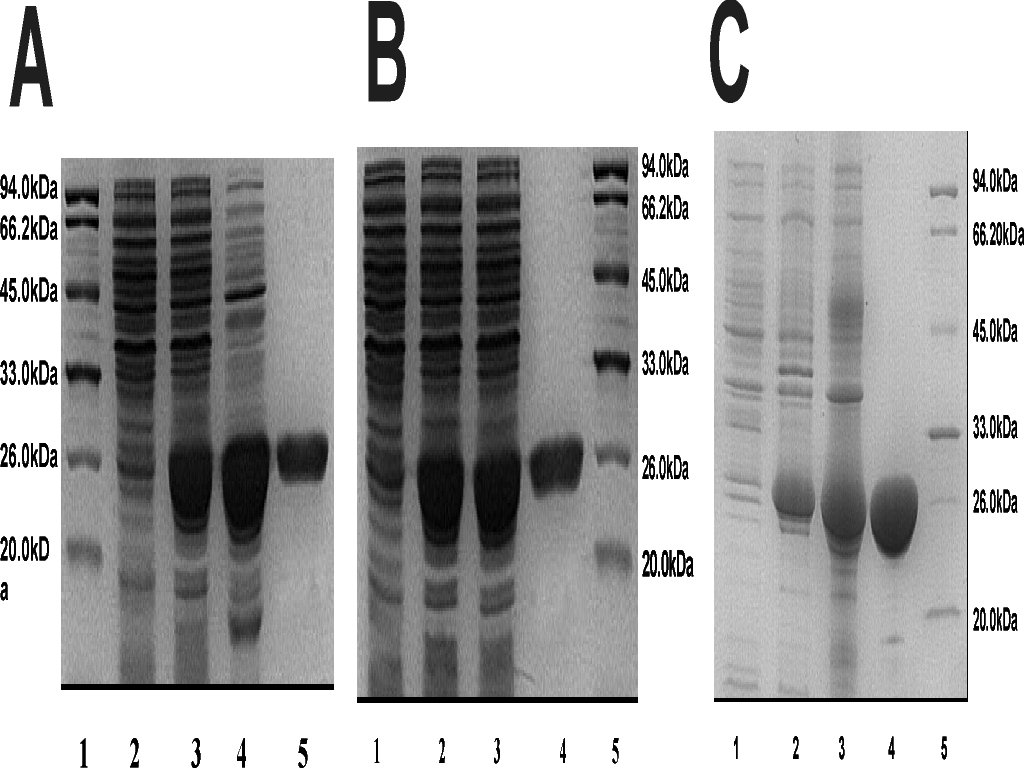
**
