# Supplementary material for: Highly Efficient Production of Soluble Proteins from Insoluble Inclusion Bodies by a Two-Step-Denaturing and Refolding Method
Source: PLoS One. 2011 Jul 29;6(7):e22981. doi: 10.1371/journal.pone.0022981 (PMC3146519; doi:10.1371/journal.pone.0022981)
Supplement: Figure S3 — The equipment of stepwise dialysis for MMP-12 refolding. (DOC) [file pone.0022981.s003.doc]

**Figure S3:** The equipment of stepwise dialysis for MMP-12 refolding

A

**Refolding buffer**

**5L**

B

**Buffer G**

**5L**

2.2 ml /min

2.2 ml /min

**C**

**5L**
