# Supplementary material for: Highly Efficient Production of Soluble Proteins from Insoluble Inclusion Bodies by a Two-Step-Denaturing and Refolding Method
Source: PLoS One. 2011 Jul 29;6(7):e22981. doi: 10.1371/journal.pone.0022981 (PMC3146519; doi:10.1371/journal.pone.0022981)

**Figure S4**: SDS-PAGE gel indicated that the catalytic domain of MMP-12 was recovered from its inclusion bodies by using double denaturing and refolding method. From right to left, lane 1: the cells before IPTG introduction; lane 2, the cells after IPTG introduction; lane 3, MMP-12 inclusion bodies; lane 4,MMP-12 in denatured buffer II ; lane 5,refolded MMP-12; lane 6, protein marker. The molecular weight of the catalytic domain of MMP-12 is 18.5KDa.


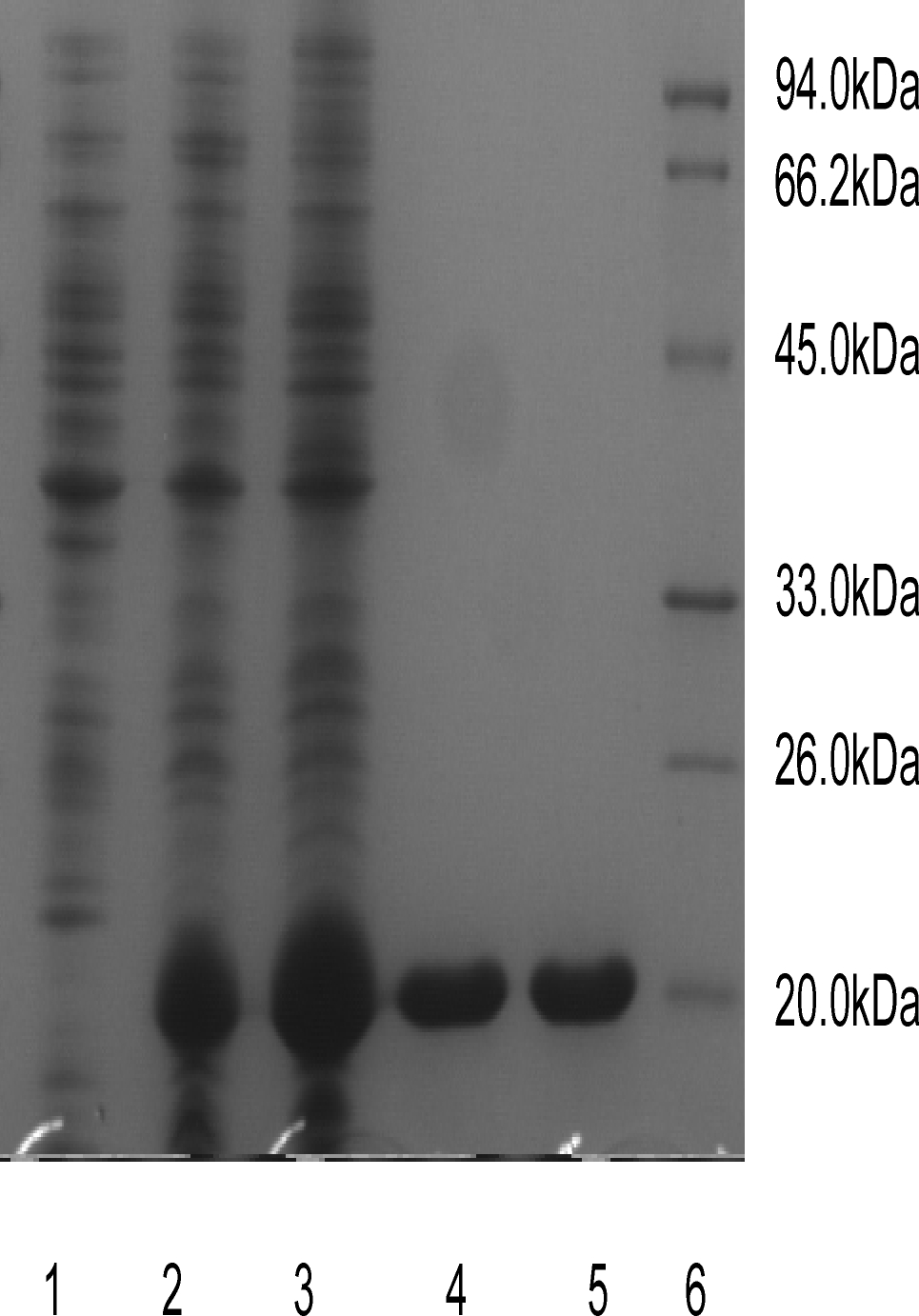

Supplement: Figure S4 — SDS-PAGE gel indicated that the catalytic domain of MMP-12 was recovered from its inclusion bodies by using double denaturing and refolding method. From right to left, lane 1: the cells before IPTG introduction; lane 2, the cells after IPTG introduction; lane 3, MMP-12 inclusion bodies; lane 4,MMP-12 in denatured buffer II ; lane 5,refolded MMP-12; lane 6, protein marker. The molecular weight of the catalytic domain of MMP-12 is 18.5KDa. (DOC) [file pone.0022981.s004.doc]
