# Supplementary material for: Highly Efficient Production of Soluble Proteins from Insoluble Inclusion Bodies by a Two-Step-Denaturing and Refolding Method
Source: PLoS One. 2011 Jul 29;6(7):e22981. doi: 10.1371/journal.pone.0022981 (PMC3146519; doi:10.1371/journal.pone.0022981)

**Figure S5:** SDS-PAGE gel indicated DBD generation through two-step denaturing and refolding. Lane 1, protein marker; lane 2, cells before IPTG induction; lane 3, cells after IPTG induction; lane 4, DBD inclusion bodies dissolved in the extracting buffer 2; lane 5, refolded DBD.


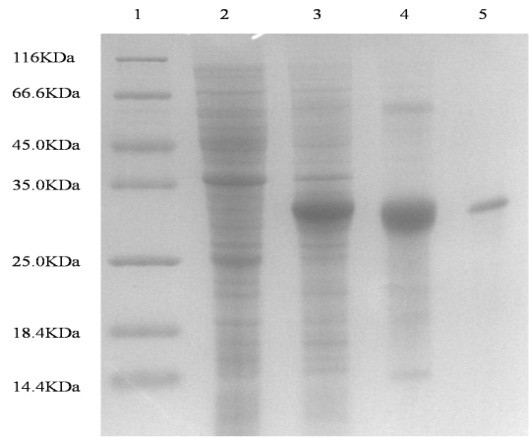

Supplement: Figure S5 — SDS-PAGE gel indicated DBD generation through two-step denaturing and refolding. Lane 1, protein marker; lane 2, cells before IPTG induction; lane 3, cells after IPTG induction; lane 4, DBD inclusion bodies dissolved in the extracting buffer 2; lane 5, refolded DBD. (DOC) [file pone.0022981.s005.doc]
