# Supplementary material for: Highly Efficient Production of Soluble Proteins from Insoluble Inclusion Bodies by a Two-Step-Denaturing and Refolding Method
Source: PLoS One. 2011 Jul 29;6(7):e22981. doi: 10.1371/journal.pone.0022981 (PMC3146519; doi:10.1371/journal.pone.0022981)
Supplement: Table S1 — The gradient dialysis buffer for refolding DBD from denatured buffer II. (DOC) [file pone.0022981.s006.doc]

**Table S1: The gradient dialysis buffer for refolding DBD from denatured buffer II.**

|  | Tris (mM) | NaCl(mM) | ZnCl_2_(mM) | glycerol(v/v) | β-mercaptoethanol (mM) | Urea(M) | pH |
| --- | --- | --- | --- | --- | --- | --- | --- |
| buffer 1 | 40 | 150 | 1 | 10% | 10 | 5 | 7.3 |
| buffer 2 | 40 | 150 | 1 | 10% | 10 | 3 | 7.3 |
| buffer 3 | 40 | 150 | 1 | 10% | 10 | 1 | 7.3 |
| buffer 4 | 40 | 150 | 1 | 10% | 10 | 0 | 7.3 |
