# Supplementary material for: Highly Efficient Production of Soluble Proteins from Insoluble Inclusion Bodies by a Two-Step-Denaturing and Refolding Method
Source: PLoS One. 2011 Jul 29;6(7):e22981. doi: 10.1371/journal.pone.0022981 (PMC3146519; doi:10.1371/journal.pone.0022981)
Supplement: Table S2 — Application of two-step denaturing and refolding technique on 88 different inclusion bodies. (DOC) [file pone.0022981.s007.doc]

**Table S2**: Application of two-step denaturing and refolding technique on 88 different inclusion bodies

| Protein Name | Accession | Organism | Yield（%） | Expression Systerm | ABS^a^ | No |
| --- | --- | --- | --- | --- | --- | --- |
| Stromal cell-derived factor 2 (SDF2) | NP_008854.2 | Homo sapiens | 100 | BL21(DE3)/PET22b | 0.003 | 1 |
| Signal transducing adaptor family member 2 | NP_060190.2 | Homo sapiens | 100 | BL21(DE3)/PET22b | 0.001 | 2 |
| C1GALT1-specific chaperone 1 | NP_689905.1 | Homo sapiens | 100 | BL21(DE3)/PET22b | 0.007 | 3 |
| Keratin 20 | NP_061883.1 | Homo sapiens | 100 | BL21(DE3)/PET22b | 0.004 | 4 |
| GDP-mannose pyrophosphorylase A (GMPPA), transcript varian1 | NP_037467.2 | Homo sapiens | 100 | BL21(DE3)/PET22b | 0.002 | 5 |
| Inositol hexaphosphate kinase 2 (IHPK2), transcript variant 1 | NP_057375.2 | Homo sapiens | 100 | BL21(DE3)/PET22b | 0.003 | 6 |
| GDNF family receptor alpha 2 (GFRA2) | NP_001486.4 | Homo sapiens | 100 | BL21(DE3)/PET22b | 0.003 | 7 |
| Fibromodulin precursor | NP_002014.1 | Homo sapiens | 100 | BL21(DE3)/PET22b | 0.002 | 8 |
| DNA-damage-inducible transcript 4 | NM_013335 | Homo sapiens | 100 | BL21(DE3)/PET22b | 0.003 | 9 |
| Deoxycytidine kinase | NP_000779.1 | Homo sapiens | 100 | BL21(DE3)/PET22b | 0.002 | 10 |
| Glucan (1,4-alpha-), branching enzyme 1 | NP_000149.2 | Homo sapiens | 100 | BL21(DE3)/PET22b | 0.002 | 11 |
| CDC42-binding protein kinase alpha isoform A | NP_003598.2 | Homo sapiens | 100 | BL21(DE3)/PET22b | 0.003 | 12 |
| Exostosin | NP_000118.2 | Homo sapiens | 100 | BL21(DE3)/PET22b | 0.001 | 13 |
| Acyl-Coenzyme A dehydrogenase, C-2 to C-3 short chain | NP_003739.2 | Homo sapiens | 100 | BL21(DE3)/PET22b | 0.002 | 14 |
| Homo sapiens Cyclin E2 isoform 3 | NP_004693.2 | Homo sapiens | 100 | BL21(DE3)/PET22b | 0.001 | 15 |
| Fructose-1,6-bisphosphatase 1 | NM_000507 | Homo sapiens | 100 | BL21(DE3)/PET22b | 0.000 | 16 |
| Homo sapiens mitochondrial ribosomal protein S22 (MRPS22) | NP_064576.1 | Homo sapiens | 100 | BL21(DE3)/PET22b | 0.002 | 17 |
| Homo sapiens prohibitin (PHB) | NP_002625.1 | Homo sapiens | 100 | BL21(DE3)/PET22b | 0.001 | 18 |
| Signal transducing adaptor family member 2 | NP_060190.2 | Homo sapiens | 100 | BL21(DE3)/PET22b | 0.003 | 19 |
| Pepsinogen 5, group I (pepsinogen A) | NP_002621.1 | Homo sapiens | 99 | BL21(DE3)/PET22b | 0.001 | 20 |
| Cyclin-dependent kinase inhibitor 1A | NP_000380.1 | Homo sapiens | 99 | BL21(DE3)/PET22b | 0.001 | 21 |
| Chromosome 16 open reading frame 61 | NM_020188 | Homo sapiens | 99 | BL21(DE3)/PET22b | 0.001 | 22 |
| hypoxia-inducible factor prolyl 4-hydroxylase (PH-4), transcript variant 2 | NM_017732 | Homo sapiens | 98.3 | BL21(DE3)/PET22b | 0.002 | 23 |
| Proteasome beta 8 subunit isoform E2 proprotein | NP_004150 | Homo sapiens | 97.9 | BL21(DE3)/PET22b | 0.007 | 24 |
| Fms-related tyrosine kinase 1 | NP_002010.2 | Homo sapiens | 97 | BL21(DE3)/PET22b | 0.003 | 25 |
| Nudix -type motif 18 | NP_079091.3 | Homo sapiens | 97 | BL21(DE3)/PET22b | 0.005 | 26 |
| Guanine monphosphate synthetase | NP_003866.1 | Homo sapiens | 89 | BL21(DE3)/PET22b | 0.010 | 27 |
| Fucosyltransferase 8 | NP_004471.3 | Homo sapiens | 85.5 | BL21(DE3)/PET22b | 0.022 | 28 |
| BTG family member 2(BTG2) | NP_006754.1 | Homo sapiens | 80 | BL21(DE3)/PET22b | 0.031 | 29 |
| Endoglin (Osler-Rendu-Weber syndrome 1) | NP_000109.1 | Homo sapiens | 74.8 | BL21(DE3)/PET22b | 0.028 | 30 |
| Cyclin D1 | NP_444284.1 | Homo sapiens | 74.3 | BL21(DE3)/PET22b | 0.041 | 31 |
| Proteasome (prosome, macropain) 26S subunit, non-ATPase, 13 | NP_002808.3 | Homo sapiens | 71.5 | BL21(DE3)/PET22b | 0.028 | 32 |
| Endothelial cell-specific molecule 1 | NP_008967.1 | Homo sapiens | 71.3/32^b^ | BL21(DE3)/PET22b | 0.065 | 33 |
| Tumor necrosis factor, alpha-induced protein 1^II^ | NP_003801.1 | Homo sapiens | 66.7/20^b^ | BL21(DE3)/PET22b | 0.461 | 34 |
| RNA (guanine-9-) methyltransferase domain containing 1 (RG9MTD1) | NP_060289.2 | Homo sapiens | 58.4/62^b^ | BL21(DE3)/PET22b | 0.564 | 35 |
| Aldehyde dehydrogenase 4A1 precursor | NP_003739.2 | Homo sapiens | 57.5/35^b^ | BL21(DE3)/PET22b | 0.386 | 36 |
| Guanine nucleotide binding protein (G protein), alpha z polypeptide | NM_002073 | Homo sapiens | 46.7/20^b^ | BL21(DE3)/PET22b | 0.152 | 37 |
| Calcyphosine | AAW27463 | Schistosoma japonicum | 100 | BL21(DE3)/PET28a | 0.002 | 38 |
| EGFP | ADQ73885.1 | Synthetic construct | 100 | BL21(DE3)/PET28a | 0.003 | 39 |
| GFP | AAN41637.1 | Aequorea coerulescens | 100 | ER2566/PTO-T7 | 0.003 | 40 |
| Ornithine decarboxylase antizyme inhibitor (OAZIN), transcript variant 2 | NP_680479.1 | Homo sapiens | 100 | BL21(DE3)/PET22b | 0.001 | 41 |
| MLF1 interacting protein | NP_078905.2 | Homo sapiens | 100 | BL21(DE3)/PET22b | 0.003 | 42 |
| Phosphorylase | NA^c^ | NA | 97 | NA | 0.003 | 43 |
| Kinetochore associated 2 | NP_006092.1 | Homo sapiens | 98 | BL21(DE3)/PET22b | 0.002 | 44 |
| Lymphotoxin | BAA00064.1 | Homo sapiens | 98 | NA | 0.001 | 45 |
| Progastricsin 2 (pepsinogen C) | NP_002621.1 | Homo sapiens | 97 | BL21(DE3)/PET22b | 0.002 | 46 |
| HSPC067 protein (HSPC067) | NP_054877.1 | Homo sapiens | 97 | BL21(DE3)/PET22b | 0.001 | 47 |
| Flotillin 2 (FLOT2) | NP_004466.2 | Homo sapiens | 96 | BL21(DE3)/PET22b | 0.006 | 48 |
| Protein phosphatase 1, regulatory (inhibitor) subunit 3B (PPP1R3B) | NP_078883.1 | Homo sapiens | 96 | BL21(DE3)/PET22b | 0.002 | 49 |
| Hypothetical protein FLJ23045 | XP_016833.1 | Homo sapiens | 95 | BL21(DE3)/PET22b | 0.006 | 50 |
| Testis derived transcript (3 LIM domains) (TES), transcript variant 1 | NM_015641 | Homo sapiens | 95 | BL21(DE3)/PET22b | 0.002 | 51 |
| G-CSF | ADI49832.1 | Homo sapiens | 95 | DH5a/ pJGW1 | 0.002 | 52 |
| Lipase | CAA00125.1 | Homo sapiens | 95 | JM109/PET22b | 0.002 | 53 |
| Nuclear prelamin A recognition factor (NARF), transcript variant 1 | NP_036468.1 | Homo sapiens | 94 | BL21(DE3)/PET22b | 0.004 | 54 |
| Cell death activator CIDE-3 | NM_016310 | Homo sapiens | 93 | BL21(DE3)/PET22b | 0.005 | 55 |
| Alpha-2-HS-glycoprotein | NP_001613.2 | Homo sapiens | 93 | BL21(DE3)/PET22b | 0.011 | 56 |
| rCs 19-2C | NA | NA | 93 | BL21(DE3)/PET28a | 0.004 | 57 |
| Progastricsin1 (pepsinogen C) | NP_002621.1 | Homo sapiens | 92 | BL21(DE3)/PET22b | 0.010 | 58 |
| V-myc myelocytomatosis viral oncogene homolog (avian) | NM_002467 | Homo sapiens | 92 | BL21(DE3)/PET22b | 0.008 | 59 |
| Centromere protein F | NP_057427.3 | Homo sapiens | 91 | BL21(DE3)/PET22b | 0.010 | 60 |
| rCs 22A | NA | NA | 91 | BL21(DE3)/PET28a | 0.001 | 61 |
| Crystallin, mu (CRYM) | NM_001888 | Homo sapiens | 90 | BL21(DE3)/PET22b | 0.001 | 62 |
| Urotensin 2 | NP_068835.1 | Homo sapiens | 90 | BL21(DE3)/PET22b | 0.001 | 63 |
| Succinate-CoA ligase, GDP-forming, beta subunit | NP_003839.2 | Homo sapiens | 90 | BL21(DE3)/PET22b | 0.005 | 64 |
| Secreted phosphoprotein 1 | NP_000573.1 | Homo sapiens | 90 | BL21(DE3)/PET22b | 0.003 | 65 |
| Neutrophil cytosolic factor 1 | NM_018044 | Homo sapiens | 90 | BL21(DE3)/PET22b | 0.001 | 66 |
| Eukaryotic translation initiation factor 2B, subunit 2 beta | NP_055054.1 | Homo sapiens | 90 | BL21(DE3)/PET22b | 0.005 | 67 |
| Transmembrane protein 11 | NM_003876 | Homo sapiens | 90 | BL21(DE3)/PET22b | 0.005 | 68 |
| ATPase, H+ transporting, lysosomal 34kDa, V1 subunit D (ATP6V1D) | NP_057078.1 | Homo sapiens | 90 | BL21(DE3)/PET22b | 0.006 | 69 |
| Dedicator of cytokinesis 5 | NM_024940 | Homo sapiens | 90 | BL21(DE3)/PET22b | 0.005 | 70 |
| Mitochondrial ribosomal protein L2 (MRPL2) | NP_057034.2 | Homo sapiens | 88 | BL21(DE3)/PET22b | 0.031 | 71 |
| Hypothetical protein FLJ22318 (FLJ22318) | NM_022762 | Homo sapiens | 85 | BL21(DE3)/PET22b | 0.009 | 72 |
| Small GTP-binding protein rab22b | AAG09690.1 | Homo sapiens | 80 | BL21(DE3)/PET22b | 0.013 | 73 |
| Elastase2b | NA | Schistosoma japonicum | 80 | BL21(DE3)/PET28b | 0.541 | 74 |
| Mitochondrial ribosomal protein L28 | NP_006419.2 | Homo sapiens | 75 | BL21(DE3)/PET22b | 0.373 | 75 |
| Rho family GTPase 1 (RND1) | AAH26356.1 | Homo sapiens | 62 | BL21(DE3)/PET22b | 0.021 | 76 |
| PP3111 protein (PP3111) | NA | NA | 58 | BL21(DE3)/PET22b | 0.373 | 77 |
| CDC20 cell division cycle 20 homolog (S. cerevisiae) | AAH12827.1 | Homo sapiens | 50 | BL21(DE3)/PET22b | 0.554 | 78 |
| Pitrilysin metallopeptidase 1 | NA | Homo sapiens | 48.4 | BL21(DE3)/PET22b | 0.501 | 79 |
| Meltalprotainase | NA | Schistosoma japonicum | 42 | BL21(DE3)/PET28a | 0.432 | 80 |
| Cs 22B | NA | NA | 30 | BL21(DE3)/PET28a | 0.897 | 81 |
| Retinol saturase (all-trans-retinol 13,14-reductase) | NP_060220.2 | Homo sapiens | 25 | BL21(DE3)/PET22b | 0.735 | 82 |
| Tumor necrosis factor (ligand) superfamily, member 10 | NP_003801.1 | Homo sapiens | 20 | BL21(DE3)/PET22b | － | 83 |
| Ankyrin repeat and SOCS box-containing 9 (ASB9) | NP_076992.1 | Homo sapiens | 20 | BL21(DE3)/PET22b | 0.897 | 84 |
| Denylosuccinate Synthetase | NA | Schistosoma japonicum | 20 | BL21(DE3)/PET28a | 0.860 | 85 |
| Trail | NP_001177871.1 | Homo sapiens | 20 | C60/ pBV220 | 0.800 | 86 |
| Pseudouridylate synthase 3 (PUS3) | NP_112597.2 | Homo sapiens | 10 | BL21(DE3)/PET22b | 0.900 | 87 |
| Plasminogen activator, urokinase | NP_002649.1 | Homo sapiens | 10 | BL21(DE3)/PET22b | 0.985 | 88 |

a) The apparent absorption at 450 nm used to indicate the turbidity for showing the aggregation extent of the proteins during refolding.

b) The ratio of overall yield of soluble proteins through two-step denaturing and refolding technique relative to that through one-step denaturing and refolding method.

c) NA Non-Available
